# A Whole-Body PSMA-PET/CT dataset with manually annotated tumor lesions

**DOI:** 10.1038/s41597-026-07821-z

**Published:** 2026-07-10

**Authors:** Katharina Jeblick, Balthasar Schachtner, Andreas Mittermeier, Jakob Dexl, Philipp Wesp, Thomas Küstner, Sergios Gatidis, Marcel Früh, Matthias P. Fabritius, Felix Herr, Lena Unterrainer, Konrad Klimek, Gabriel Sheikh, Guido Böning, Matthias Brendel, Jens Ricke, Rudolf A. Werner, Sijing Gu, Lalith Kumar Shiyam Sundar, Michael Ingrisch, Thomas Geyer, Clemens Cyran

**Affiliations:** 1 Department of Radiology, LMU University Hospital, LMU Medizin, Ludwig-Maximilians-Universität München, Munich, Germany; 2 Comprehensive Pneumology Center (CPC-M), Member of the German Center for Lung Research (DZL), Munich, Germany; 3 Munich Center for Machine Learning (MCML), Munich, Germany; 4 University Hospital Tübingen, Department of Diagnostic and Interventional Radiology, Medical Image and Data Analysis (MIDAS.lab), Tübingen, Germany; 5 Department of Nuclear Medicine, LMU University Hospital, LMU Medizin, Ludwigs-Maximilians-Universität München, Munich, Germany

## Abstract

We describe a publicly available, large, annotated dataset of 597 whole-body Positron Emission Tomography/Computed Tomography (PET/CT) studies with Prostate-Specific Membrane Antigen (PSMA)-targeting radiotracers ([18 F]PSMA and [68Ga]Ga-PSMA-11) from 378 male patients with suspected or diagnosed prostate carcinoma. Scans were acquired between 2014 and 2022 on three clinical PET/CT scanners. The imaging protocol consisted of PET and diagnostic CT acquisitions extending from the skull base to the mid-thigh. All PSMA-expressing tumor lesions were manually segmented on the PET images in 3D space using dedicated software. The dataset includes anonymized DICOM files of all PET/CT studies, corresponding DICOM segmentation masks, and a TSV file with patient age, PET/CT manufacturer and model name, PET radionuclide, and information on whether CT contrast agent was used. We demonstrate how this dataset can be used for deep learning-based automated analysis of PET/CT. Together with a previously published whole-body Fluorodeoxyglucose (FDG)-PET/CT dataset, this dataset was provided in the Medical Image Computing and Computer Assisted Intervention Society (MICCAI) registered autoPET III and IV Grand Challenges to enable the development of multi-tracer machine learning models for automated lesion segmentation in whole-body PET/CT.

## Background & Summary

Positron Emission Tomography/Computed Tomography (PET/CT) has evolved into an integral diagnostic modality in oncological therapy guidance^1^. In this hybrid imaging concept, CT provides high-resolution 3D anatomical information. Radiotracer-enhanced PET contributes functional and molecular information on tissue metabolism and tumor biology^2^. Fluorodeoxyglucose (FDG) labeled with ^18^F is the most widely used PET radiotracer to visualize increased glucose metabolism of malignant tumor cells known as the Warburg effect. Due to its superior diagnostic accuracy compared to conventional staging techniques, [^18^F]FDG PET/CT is recommended by international guidelines^3–5^ for staging of different tumor entities, among them lung cancer, lymphoma, and melanoma. However, well-documented limitations of [^18^F]FDG exist with regard to its low diagnostic accuracy, particularly in highly differentiated tumors, such as highly differentiated neuroendocrine tumors, hepatocellular carcinoma, and prostate cancer. Selective radiotracers have been developed to address these limitations, among them ^68^Ga and ^18^F-labeled PSMA (prostate-specific membrane antigen) targeting ligands for imaging of prostate carcinoma. Although not specific for prostate cancer, PSMA is overexpressed in the healthy prostate by a factor of approximately 100 compared to most other body tissues. By prostate carcinoma cells it is overexpressed by a factor of approximately 1000^6,7^. Therefore, PSMA-targeting radiotracers demonstrate a superior tumor-to-background ratio allowing for the sensitive detection of small lymph node and bone metastases with previously unknown diagnostic accuracy^8^. PET/CT with ^68^Ga and ^18^F-labeled PSMA ligands is recommended by international guidelines^9^ for the diagnostic workup in different clinical scenarios along the prostate cancer patient journey, among them biochemically recurrent prostate cancer and patient assessment during PSMA-targeting radioligand therapy. Furthermore, PSMA theranostics has become a central paradigm in metastatic prostate cancer. In this setting, [68Ga]Ga-PSMA PET/CT serves as the diagnostic counterpart to [177Lu]Lu-PSMA radioligand therapy, and guides staging, response assessment, and treatment selection^9^.

Patients with metastatic prostate cancer frequently present with a high tumor burden in lymph nodes and bone. Quantitative assessment of individual lesions on PSMA-targeting PET/CT can therefore be tedious. As a result, clinical PET/CT reports usually provide a predominantly qualitative assessment of tumor lesions and therapy response with (semi-) quantitative measurements of size and PSMA expression in exemplary lesions. Several studies^10–13^, however, have demonstrated that a full quantitative analysis of PET/CT enables more precise and individualized therapeutic decisions with potentially beneficial effects on patient outcomes. Furthermore, PSMA PET/CT is increasingly being explored for quantitative theranostic applications, including automated whole-body tumor segmentation for lesion characterization and pre-therapy dosimetry prediction^14,15^.

While manual segmentation of all metastatic lesions is too time- and resource-intensive for routine clinical use, automated tumor segmentation in PSMA-targeted PET/CT could facilitate translation of quantitative assessment into clinical practice. It could improve prognostication and therapy response assessment, and future theranostic applications such as enhanced dose prediction^14^ and individualized treatment planning in patients undergoing [177Lu]Lu-PSMA therapy^14,15^. However, until a few years ago, mainly semi-automated tools^16,17^ were available for quantitative image analysis and tumor segmentation, necessitating additional manual adjustment.

Artificial intelligence offers the potential for fully automated quantitative segmentation of PET/CT images with different radiotracers. Previous studies^18–24^ have demonstrated the effectiveness of AI in detecting and segmenting metabolically active lesions in whole-body FDG-PET/CT. Our prior work on open-sourcing a large, manually annotated dataset^25,26^ with FDG-PET/CT studies of patients with lung cancer, lymphoma, and melanoma effectively served as a catalyst for the development of state-of-the-art AI algorithms for automated quantitative FDG-PET/CT image analysis in the context of the MICCAI autoPET I and II Grand Challenge^27,28^. In the case of PSMA-PET/CT, the development of AI models for automated quantitative image analysis however trailed behind. One of the primary reasons was the scarcity of large, publicly accessible PSMA-PET/CT datasets with image-level reference labels that comprehensively capture heterogeneous metastatic burden, essential for the development and validation of robust AI models.

To enable automated quantitative image analysis for PSMA-targeting PET/CT in prostate cancer, we provide a publicly available dataset of 597 PSMA-PET/CT examinations ([18 F]PSMA and [68Ga]Ga-PSMA-11 PET/CTs) from 378 patients with suspected or diagnosed prostate cancer, together with voxel-wise manual lesion annotations. In addition, we present a vanilla nnU-Net baseline model trained on this dataset. Data preparation and labeling followed the methodology of the published FDG-PET/CT dataset^25,26^. Our dataset is the first and, to date, the largest publicly available PSMA-PET/CT dataset.

## Methods

Publication of anonymized retrospective data was approved by the institutional review board (Ethics Committee, Medical Faculty, LMU Munich; application numbers 21-1230, DRKS-Nr. 00026991, and 21-0098, DRKS-Nr. 00024094), as well as by the institutional data security and privacy review board. As this retrospective study used fully anonymized, non-identifiable imaging data, the requirement for individual informed consent was waived by the institutional review board.

### Data collection

**Fig. 1:**
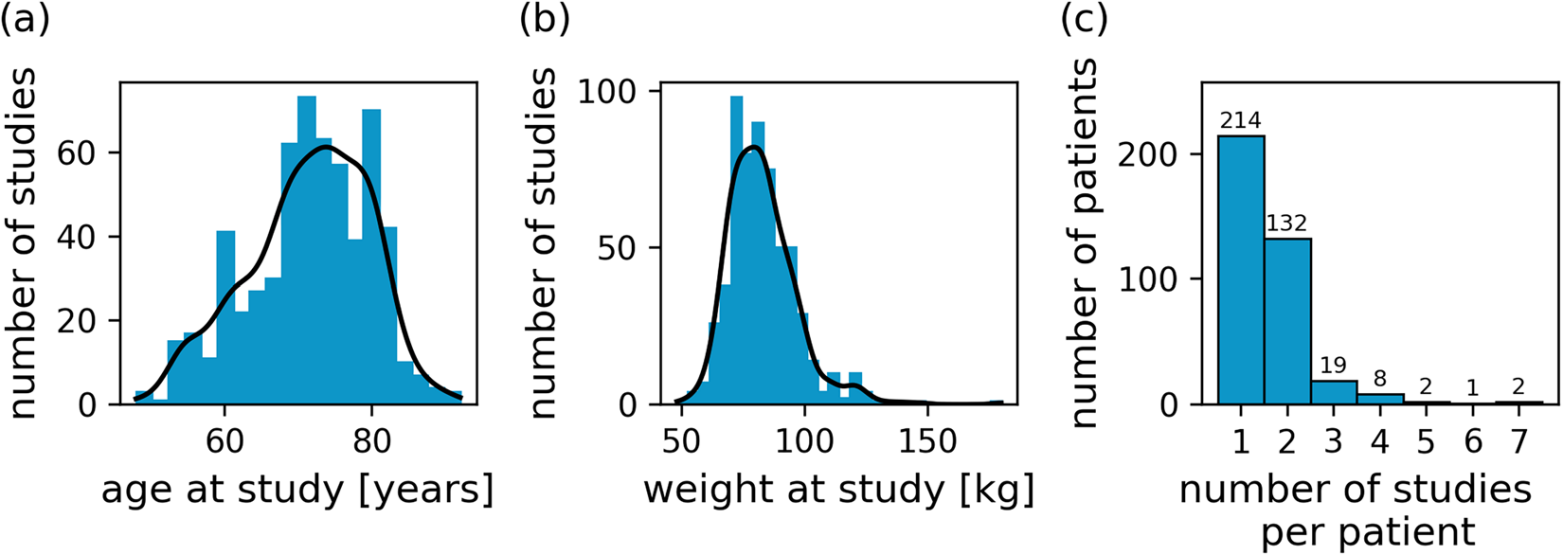
(**a**) Histogram of patient age, and (**b**) patient weight at PSMA-PET/CT acquisition, and (**c**) number of PSMA-PET/CT studies per patient.

This retrospective dataset includes 378 male patients with diagnosed or suspected prostate carcinoma treated at LMU University Hospital Munich. It comprises 597 whole-body [^18^F]PSMA-1007 and [^68^Ga]Ga-PSMA-11 PET/CT examinations extending from the skull base to the mid-thigh, denoted as whole-body PET/CT hereafter. PSMA-PET/CT examinations were performed for primary staging, therapy monitoring, and recurrence detection as part of the clinical routine between 2014 and 2022. Of these 597 examinations, 539 are positive samples with at least one PSMA-avid tumor lesion, and 58 are negative samples without any PSMA-expressing tumor lesion. Negative samples originate from patients who underwent PET/CT (e.g., for restaging) after successful therapy. Figure 1 provides a summary of patient characteristics. Patients were 71 ± 8 years (range: [48, 92] years) and weighed 83 ± 14 kg (range: [48,180] kg) on average ± SD at PSMA-PET/CT acquisition. Individual patients included in the dataset received between 1 and 7 PSMA-PET/CT examinations at LMU University Hospital Munich during the course of treatment (with a median of 1 study and an interquartile range (IQR) of [1, 2] studies per patient).

### PET/CT acquisition

**Fig. 2:**
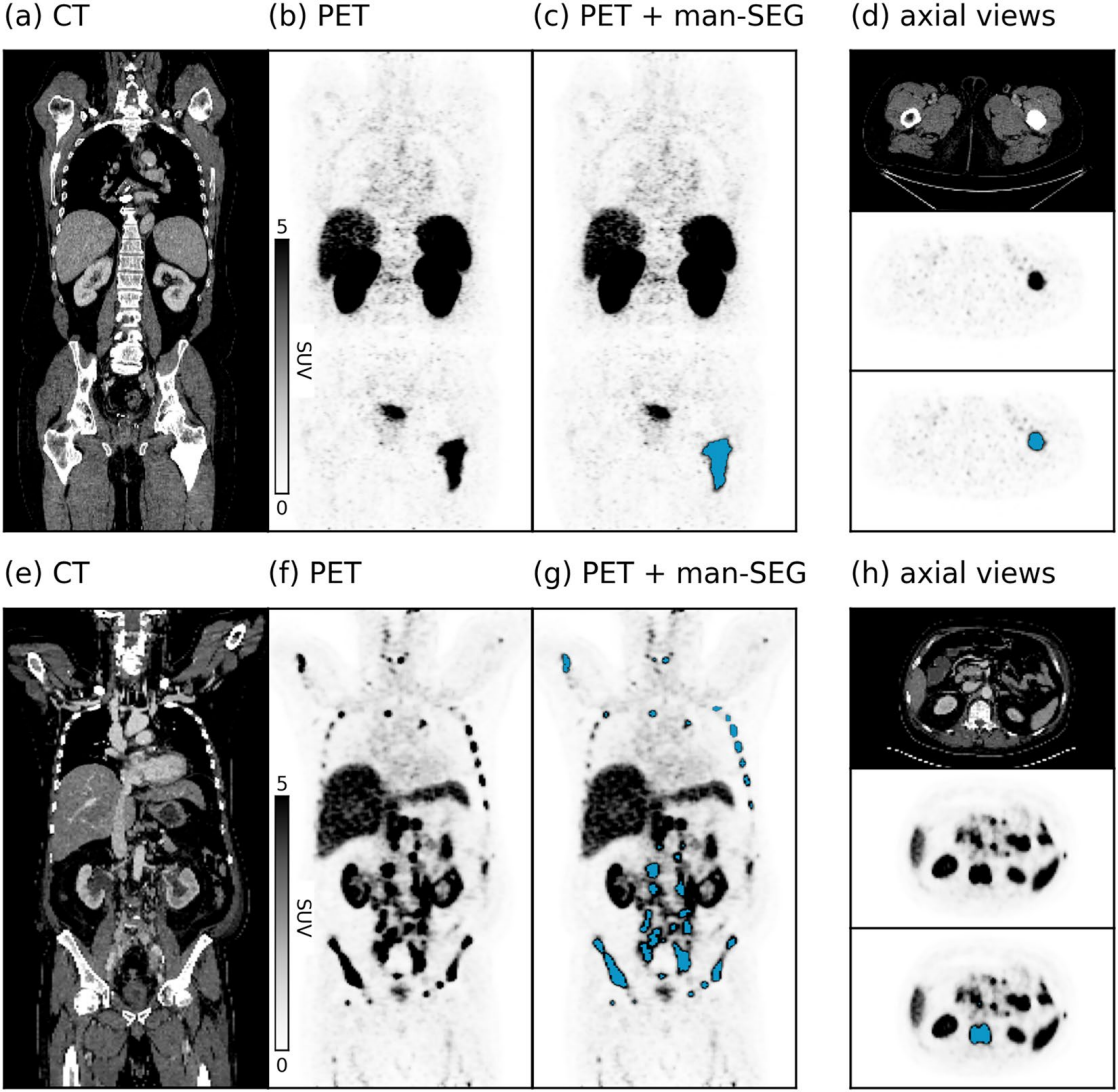
PSMA-PET/CT with (**a-d**) one pathological tumor lesion ([^68^Ga]Ga-PSMA-11) and (**e-h**) 197 pathological tumor lesions ([^18^F]PSMA-1007). (**a,e**) CT coronal view. (**b,f**) PET coronal view. (**c,g**) PET coronal view with manual tumor segmentations (man-SEG, blue overlay). (**d,h**) Axial views of CT, PET, and PET with manual tumor segmentations (blue overlay).

PET/CT examinations were acquired in accordance with international guidelines^29^ for tumor imaging on three different scanners, jointly operated by the Department of Nuclear Medicine and the Department of Radiology: GE Discovery 690 (230 studies), Siemens Biograph mCT Flow 20 (251 studies), Siemens Biograph 64-4 R TruePoint (116 studies). An overview of the data with acquisition parameters is given in Table 1. Example PET/CT images are displayed in Fig. 2.

**Table 1:** Data overview with acquisition parameters per scanner and in total. Numbers represent study counts unless referring to patients or specified otherwise.

PET/CT scanner		GE Discovery 690	Siemens Biograph mCT Flow 20	Siemens Biograph 64-4 R TruePoint	total
patients*		172	178	94	378
**studies**		230	251	116	597
with lesions		196	237	106	539
without lesions		34	14	10	58
**PET**					
**radionuclide**	**total dose** (mean ± SD [MBq])				
^18^F	246 ± 27	127	219	23	369
^68^Ga	214 ± 45	103	32	93	228
**resolution**					
slice thickness ([mm])		3.27	3.00	5.00	
voxel size ([mm^3^])		2.73 × 2.73 × 3.27**	4.07 × 4.07 × 2.00	4.07 × 4.07 × 5.00	
**CT**					
**contrast agent**					
yes		216	246	109	571
no		14	5	7	26
**resolution**					
slice thickness ([mm])		2.50 (227 studies); 5.00 (3 studies)	3.00	3.00	
voxel size ([mm^3^])		0.98 × 0.98 × 2.50 (225 studies); 0.98 × 0.98 × 5.00 (3 studies); 0.98 × 0.98 × 3.22 ± 2.89 (1 study)**; 1.15 × 1.15 × 2.50 (1 study)	0.98 × 0.98 × 3.00 (248 studies); 1.52 × 1.52 × 2.00 (3 studies)	0.98 × 0.98 × 3.00	

*Some patients underwent follow-up PET/CT examinations on different scanner types. **One study had an unequal PET slice spacing (mean ± SD) of 4.47 ± 4.24 mm and CT slice spacing (mean ± SD) of 3.22 ± 2.89.

PSMA-ligands ([^18^F]PSMA-1007, [^68^Ga]Ga-PSMA-11) were injected via intravenous bolus. ^18^F was used in 369 examinations, ^68^Ga in 228 examinations. Patients received a total dose of 246 ± 27 MBq (range: [110, 341] MBq) for ^18^F-labeled ligands and 214 ± 45 MBq (range: [86, 324] MBq) for ^68^Ga-labeled ligands on average ± SD, respectively. Whole-body PET/CT acquisitions were started post-injection after an average ± SD uptake time of 74 ± 22 minutes (range: [6, 181] minutes) for ^18^F-labeled and 74 ± 19 minutes (range: [7, 195] minutes) for ^68^Ga-labeled ligands. Patients were positioned supine with both arms elevated above the head. PET/CT scans were conducted from the skull base to the mid-thigh for the diagnostic CT and vice versa for the PET images. The following scan parameters were used for the diagnostic CT scans: reference tube current exposure time product of 143 ± 58 mAs on average ± SD (range: [22, 321] mAs); tube voltage of median 120 kV and IQR of [100, 120] kV (range: [80, 140] kV). Weight-adapted intravenous CT contrast agent was administered (571 studies) according to the standard protocol^30^ of 1.5 ml per kg of body weight (up to a maximum of 120 ml), unless contraindications were present (26 studies). CT data were reconstructed with standard, vendor-provided image reconstruction algorithms in transverse orientation with a slice thickness of 2.50 mm for GE Discovery 690 (except for 3 cases with 5.00 mm), and a slice thickness of 3.00 mm for Siemens Biograph mCT Flow 20 and Biograph 64-4 R TruePoint. Voxel size was 0.98 × 0.98 × 3.00 mm³ for Siemens Biograph mCT Flow 20 (248 studies) and Biograph 64-4 R TruePoint (116 studies), 0.98 × 0.98 × 2.50 mm³ for GE Discovery 690 (225 studies), 1.52 × 1.52 × 2.00 mm³ for Siemens Biograph mCT Flow 20 (3 studies), and 0.98 × 0.98 × 5.00 mm³ (3 studies), 1.15 × 1.15 × 2.50 mm³ (1 study), 0.98 × 0.98 × 3.22 ± 2.89 mm³ with unequal slice spacing (mean ± SD) for GE Discovery 690.

PET acquisition was performed in 3D mode. PET data were reconstructed with attenuation correction derived from corresponding CT data. For GE Discovery 690 the reconstruction process employed a VPFX algorithm with a voxel size of 2.73 × 2.73 × 3.27 mm³ (except for one study with unequal slice spacing (mean ± SD) of 4.47 ± 4.24 mm instead of 3.27 mm), for Siemens Biograph mCT Flow 20 a PSF + TOF algorithm (2 iterations, 21 subsets) with a voxel size of 4.07 × 4.07 × 2.00 mm³, and for Siemens Biograph 64-4 R TruePoint a PSF algorithm (3 iterations, 21 subsets) with voxel size 4.07 × 4.07 × 5.00 mm³. Except for the GE Discovery 690 study with unequal slice spacing, the PET slice thickness was equal to the slice spacing.

### Data labeling

The primary tumor and/or all metastases were manually segmented by a medical imaging expert (S.G. with 3 years of experience in hybrid imaging) and cross-checked by board-certified medical imaging experts with 4 years and > 10 years of experience in hybrid imaging using specialized CE-certified software (Mint Lesion™, Mint Medical, Heidelberg, Germany). The attenuation-corrected PET volumes with voxel values converted to standardized uptake values (SUV, see Eq. (1) in Section Usage Notes) and a corresponding CT were displayed side by side or as an overlay. Tumor lesions with significantly increased PSMA expression were segmented in 3D space by drawing circular volumes of interest, in which voxels with SUV values above a user-defined threshold were pre-segmented automatically and then manually corrected slice by slice, resulting in 3D binary segmentation masks. The threshold was used to accelerate the manual annotation process and was not kept fixed but adapted individually for the images based on visual inspection. Examples of manually segmented tumor lesions are presented in Fig. 2(c,d,g,h).

### Data selection and processing

After annotation, all image data (full studies) in DICOM format and manual segmentation masks in NRRD (nearly raw raster data) format were exported from the annotation software. In the data selection process, the attenuation-corrected PET scan and the most suitable CT series for diagnostic assessment were selected, typically the contrast-enhanced CT with the highest number of slices.

For each patient and study, patient age (in years), the manufacturer and manufacturer model name, the PET radionuclide, and information (yes/no) about the use of CT contrast agent were extracted from the DICOM headers and collected in a metadata file. The information on radionuclides and the use of CT contrast agents was additionally visually validated by a medical imaging expert with 10 years of experience in hybrid imaging.

For each study, individual manual lesion segmentation files were merged into a single segmentation mask, matching the PET image volume and voxel size, and converted to a DICOM SEG using the highdicom package v0.22.^31^ with Python v3.8.13. DICOM tags of PET and CT volumes, as well as corresponding segmentation masks, were anonymized upon data upload to The Cancer Imaging Archiv^32^ using their CTP Submission Wizard. In line with recent NIH policy changes, TCIA applied an additional routine defacing procedure to DICOM images, despite scans being limited to the skull base.

## Data Records

This dataset is publicly accessible in DICOM format on The Cancer Imaging Archive (TCIA) under the collection name *PSMA-PET-CT-Lesions*^33^ (https://www.cancerimagingarchive.net/collection/PSMA-PET-CT-Lesions/). The FDAT Research Data Repository of the University Tübingen, Germany, also provides the dataset in NIfTI format^34,35^. DICOM data was additionally de-faced by TCIA, while NIfTI data was unaltered. Further, two versions with slightly different segmentation masks are currently available. The data description in this manuscript is based on the TCIA dataset^33^ Version 2. For quantitative PSMA-PET/CT image analysis and the nnU-Net baseline model we used the data in NIfTI format^35^.

### DICOM data

**Fig. 3:**
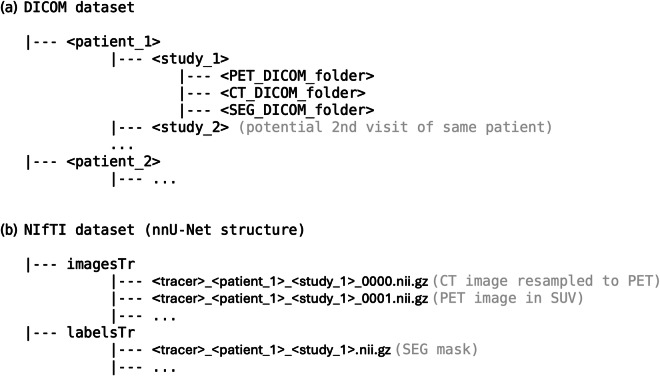
Dataset structure. Patients are identified by a unique, anonymized ID. (**a**) TCIA DICOM dataset: Each study folder contains three subfolders with DICOM files of the PET volume, the CT volume, and the segmentation mask. (**b**) NIfTI dataset in nnU-Net structure: The DICOM dataset can be converted to NIfTI format in nnU-Net structure with the provided conversion repository, with CT images resampled to the size and resolution of the PET volumes.

The PSMA-PET/CT dataset comprises 378 patients with a total of 597 studies. Each study includes three image series stored in DICOM format: a whole-body PSMA-PET volume, a corresponding whole-body CT volume, and a binary segmentation mask stored as a DICOM Segmentation Object. For segmentation masks, the series description tag specifies whether tumor lesions are present, with entries labeled “Segmentation – Tumor lesions detected” or “Segmentation – No tumor lesions detected”. In total, the dataset contains 1,791 image series, corresponding to 374,151 individual DICOM files (total size of approximately 190 GB). The hierarchical directory structure follows the same convention as the previously published FDG-PET/CT DICOM dataset^25,26^ as illustrated in Fig. 3(a). Each patient is uniquely identified by an anonymized patient ID.

### Metadata

In addition to the imaging data, a metadata file in Tab-Separated Values (TSV) format is provided for each patient and study. This file contains information about patient age (in years), PET/CT manufacturer and model name, PET radionuclide, and use of CT contrast agent (yes/no). Additional metadata, including patient body weight, injected dose, uptake time, and PET/CT acquisition parameters, are stored within the corresponding DICOM tags of the imaging data.

## Data Overview

**Fig. 4:**
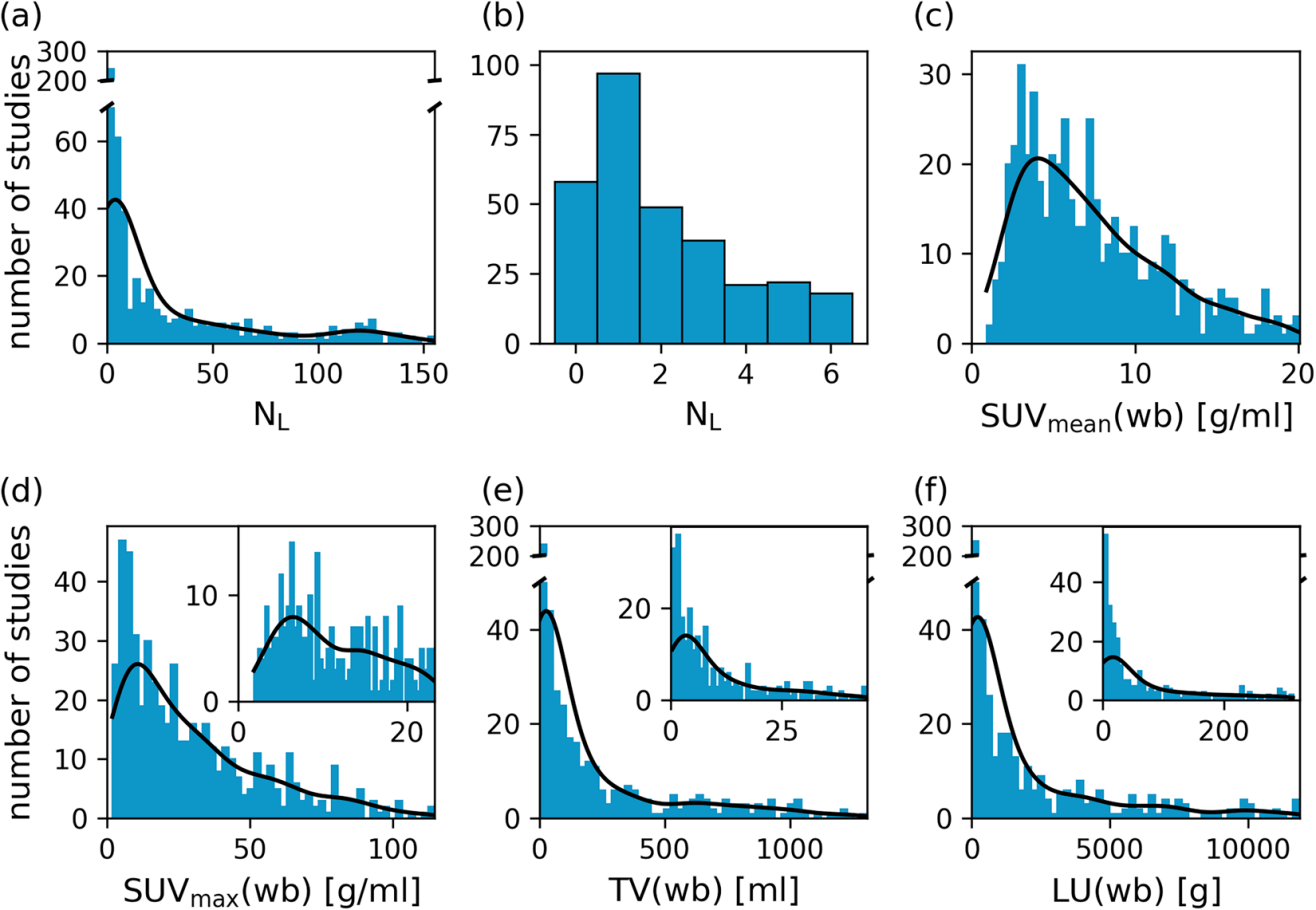
Overall (**a**) and zoomed-in (**b**) distribution of total number of lesions per study, ${N}_{L}$, and (**c-f**) distribution of semi-quantitative parameters for studies with PSMA-avid lesions: (**c**) whole-body mean SUV, ${SU}{V}_{{mean}}({wb})$, (**d**) whole-body maximum SUV, ${SU}{V}_{\max }({wb})$, (**e**) whole-body tumor volume, ${TV}({wb})$, and (**f**) whole-body lesion uptake, ${LU}({wb})$. Distributions are plotted for the 95% range of values for improved visualization. Insets in (**d-f**) show zoomed-in distributions up to respective median values.

Figure 4 summarizes key parameters of quantitative PSMA-PET/CT image analysis. Figure 4(a) shows the overall distribution of the total number of lesions, ${N}_{L}$. A single lesion was defined as a connected component with an 18-connectivity^36^. The median number of PSMA-avid lesions per positive study in this dataset is 6, with an IQR of [1, 41] lesions (range: [0, 318]). Figure 4(b) details the number of studies for each lesion number up to the median of 6 lesions per study. Figure 4(c) and Fig. 4(d) illustrate commonly used clinical metrics derived from standardized uptake values (SUV): the distribution of whole-body (wb) mean SUV, ${SU}{V}_{{mean}}({wb})$, and whole-body maximum SUV, ${SU}{V}_{\max }({wb})$, for positive studies, respectively. On average ± SD, we found a median ${SU}{V}_{{mean}}({wb})$ of 6.74 g/ml with IQR [3.92, 11.00] g/ml (range: [0.90, 64.83] g/ml) and a median ${SU}{V}_{\max }\,({wb})$ of 23.29 g/ml with IQR [9.62, 48.25] g/ml (range: [1.87, 392.79] g/ml). Figure 4(e) displays the distribution of the whole-body tumor volume ${TV}({wb})$ for positive studies with a median of 44.08 ml and an IQR of [5.87, 247.59] ml (range: [0.41, 2885.14] ml). Figure 4(f) shows the distribution of the whole-body lesion uptake, ${LU}({wb})$, per positive study, with a median ${LU}({wb})$ of 325.25 g and an IQR of [26.09, 2365.18] g (range: [1.13, 23226.17] g).

## Technical Validation

As a technical validation and use-case scenario, we trained and evaluated a vanilla 3D full-resolution nnU-Net model (v2). The nnU-Net framework^37^ is a standardized and widely adopted deep learning pipeline with automated preprocessing and adaptive configuration for medical image segmentation. The network, based on a 3D U-Net architecture, was trained on a custom split of the PSMA-PET/CT dataset with 5-fold cross-validation. PET volumes were standardized to SUV units (using Eq. (1) in Section Usage Notes), and corresponding CT volumes were resampled to match the PET resolution. SUV and resampled CT volumes were provided as input channels, while the reference segmentation masks served as labels (Fig. 3(b)). Training was performed on a single NVIDIA A100 (40 GB) using pre-configured hyperparameters (maximum of 1,000 epochs, initial learning rate of 0.01 with polynomial decay).

To assess segmentation performance, we applied three evaluation metrics introduced in the MICCAI autoPET Grand Challenge^27^, tailored to the task of tracer-avid lesion segmentation in PET/CT: the foreground Dice similarity coefficient (DSC) measuring lesion overlap, the false positive volume (FPV), measuring the volume of predicted connected components without overlap to reference segmentations, and the false negative volume (FNV), measuring the volume of reference connected components not captured by the prediction. For cases without annotated tumor lesions, only FPV was considered. These metrics address the difficulty of distinguishing (small) tumor lesions from organs with physiologically high PSMA uptake (including lacrimal and salivary glands, liver, spleen, and gastrointestinal tract, as well as radionuclide-specific uptake patterns, such as renal parenchyma for ^18^F tracers, and urinary tract activity for ^68^Ga tracers, along with variable uptake in bone marrow). In addition, we calculated lesion-level detection metrics, namely recall, precision, and F1 score^38^. Lesion detection was assessed using an overlap-based matching criterion between predicted and reference lesions. Lesions were considered detected if they exhibited a non-zero overlap (IoU threshold corresponding to at least one overlapping voxel). Multiple predictions were allowed to match the same reference lesion.

**Fig. 5:**
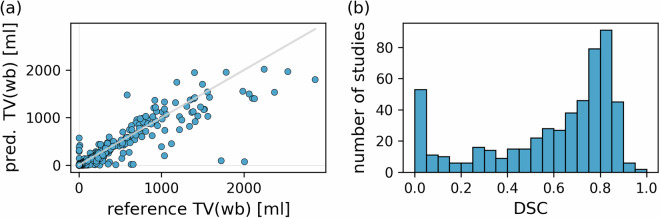
Quantitative evaluation of automated tumor lesion segmentation. (**a**) Automatically predicted versus reference whole-body tumor volume ${TV}({wb})$ across all studies. For a few cases without annotated lesions large tumor volumes were predicted. Closer inspection confirmed these predictions to be correct, revealing lesions missed during manual annotation and highlighting the potential of automated segmentation. (**b**) Distribution of Dice Similarity Coefficients (DSC) for automated versus manual tumor segmentation in positive studies.

**Fig. 6:**
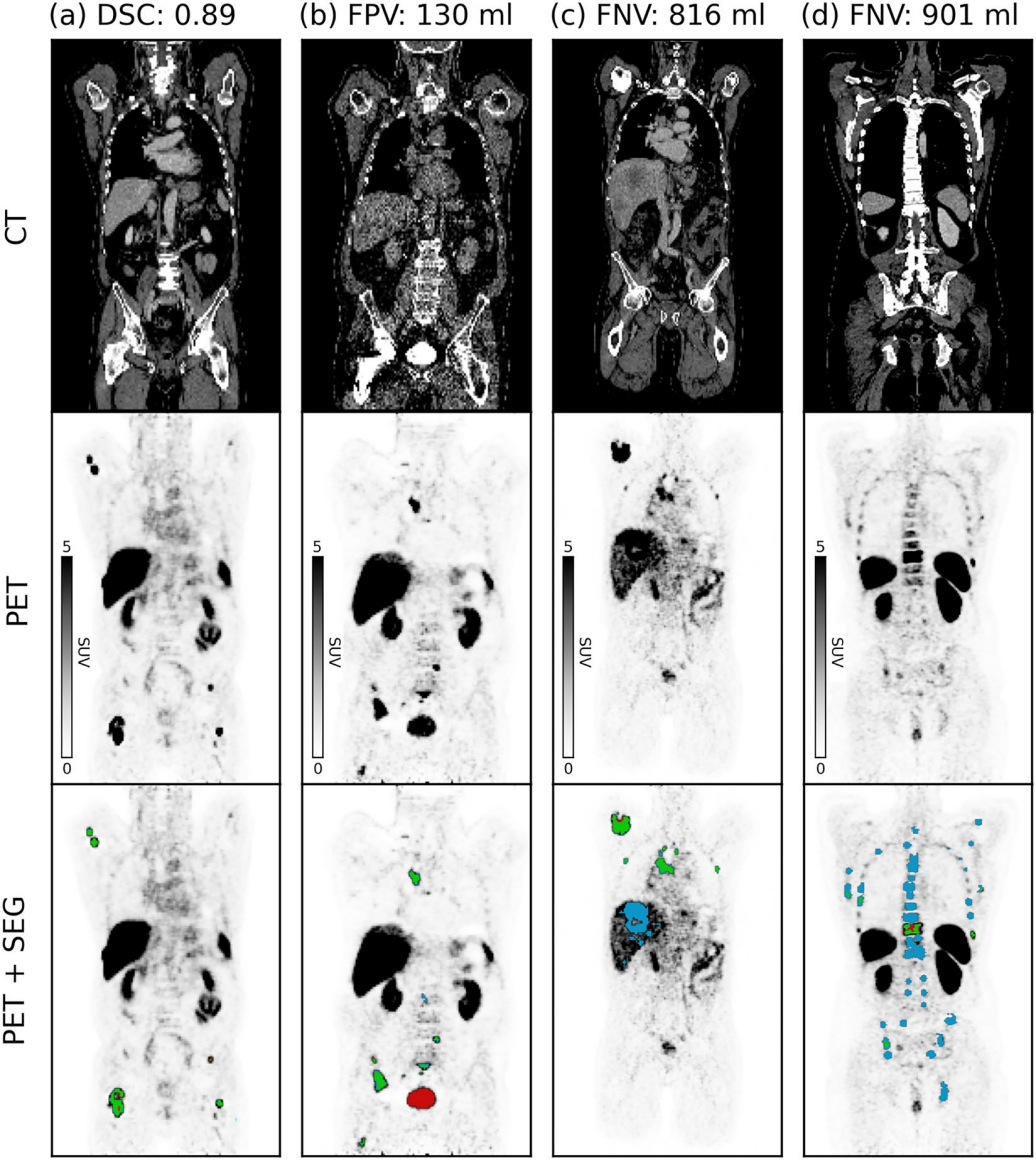
Qualitative examples of automated tumor lesion segmentation results, illustrating (**a**) accurate predictions and (**b–d**) cases with larger mis-segmented regions. Each column shows coronal views of the CT (top row), PET (middle row), and PET with reference and predicted segmentations overlaid (bottom row). Green overlay: true positive predicted voxels. Blue overlay: false negative predicted voxels. Red overlay: false positive predicted voxels. (**a**) Example with high Dice Similarity Coefficients (DSC), i.e., strong agreement between predicted and reference tumor lesions. (**b**) Example with high FPV due to incorrect segmentation of physiological uptake in urinary bladder. (**c,d**) Examples with marked FNV: (**c**) missed large liver tumor lesion confounded by physiological uptake, and (**d**) missed treated bone metastases with lower SUV.

The trained vanilla nnU-Net demonstrated overall solid performance (Fig. 5). The Pearson correlation of 0.91 (p < 0.001) indicates that, globally, automated tumor burden estimation is well aligned with the reference (Fig. 5(a)). At the voxel level, segmentation performance was still reasonable with a median DSC of 0.70 (IQR [0.44–0.81]) and a mean (±SD) DSC of 0.59 (±0.28) on positive samples (Fig. 5(b)), but lower as the mean (±SD) DCS of 0.73 (±0.23) of the vanilla nnU-Net baseline model for the FDG-PET/CT dataset^25^. Median FNV and FPV were low (1.64 ml, IQR [0, 7.83] ml, and 1.30 ml, IQR [0, 5.08], respectively), indicating that most lesions were correctly captured and few spurious regions were predicted. Mean (±SD) values were 17.42 ± 71.09 ml for FNV and 10.90 ± 54.33 ml for FPV, and only slightly higher than for the FDG-PET/CT nnU-Net baseline model. However, the wide distribution of DSC, FNV, and FPV highlights variability across patients, with some cases showing larger areas of missed lesions or false positives (Fig. 6). Median lesion-level recall, precision and F1 score resulted in 0.91 (IQR [0.67,1.00]), 0.80 (IQR [0.50, 0.96]), and 0.81 (IQR [0.58, 0.90]), respectively.

Apart from the presented use case, the potential of the PSMA-PET/CT dataset for automated PET/CT image analysis has been extensively demonstrated through the autoPET III (https://autopet-iii.grand-challenge.org/) and IV (https://autopet-iv.grand-challenge.org/) Grand Challenges for automated lesion segmentation in whole-body PET/CT, hosted at MICCAI. In these challenges, the previously published FDG-PET/CT dataset^25,26^ and the PSMA-PET/CT dataset were jointly provided as training data. AutoPET III focused on refining fully automated lesion segmentation across multi-tracer and multi-center settings^38^. This was the first large-scale challenge to incorporate PSMA data, and it led to the development of numerous state-of-the-art algorithms^39–41^. AutoPET IV, in turn, explored an interactive human-in-the-loop approach to enhance lesion segmentation performance in clinical scenarios. Looking ahead, future applications of this dataset may include automated lesion tracking on whole-body PET/CT for clinical tasks such as treatment response monitoring.

## Usage Notes

### autoPET challenge dataset

Version 1 of the PSMA-PET/CT dataset together with the FDG-PET/CT dataset^25,26^ were provided (in NIfTI format) as training data in the MICCAI-registered autoPET III (https://autopet-iii.grand-challenge.org/) and autoPET IV (https://autopet-iv.grand-challenge.org/) Grand Challenges.

### Image viewing and analysis

To view the DICOM data, we recommend open-source medical image data viewers such as 3D Slicer (https://www.slicer.org/) or the Medical Imaging Interaction Toolkit (https://www.mitk.org/). For computational analysis, e.g., in Python, 3D image volumes can be read using open-source libraries such as pydicom (https://pydicom.github.io/), nibabel (https://nipy.org/packages/nibabel/index.html), or SimpleITK (https://simpleitk.org/).

### Image preprocessing and format conversion

To facilitate data utilization, we recommend converting the PSMA-PET/CT dataset from DICOM to other widely used medical image formats, preferably NIfTI, but also mha or hdf5 if required. For conversion to NIfTI format in the nnU-Net structure (as illustrated in Fig. 3(b)) and to reproduce the autoPET preprocessing, we recommend using the repository https://github.com/ClinicalDataScience/tcia-psma-pet-ct-preprocessing. These Python scripts perform DICOM-to-NIfTI conversion, resample CT volumes to match the size and resolution of the corresponding PET volume, and normalize PET voxel values to standardized uptake values (SUV) based on body mass.

In PSMA-PET/CT imaging, SUV provides a standardized, quantitative measure of tracer uptake, enabling inter-patient and inter-scan comparison of PSMA activity^9^. It is frequently used for tumor characterization and treatment response monitoring in prostate cancer. For a voxel $v$, SUV (normalized to body mass) is calculated by weighting the corresponding radioactivity concentration (PET value of voxel $v$) with the injected dose of the tracer and the patient’s body weight:

1 $${SUV}(\nu )[g/{ml}]=\frac{{tissue\; radioactivity\; concentration}(\nu )[{Bq}/{ml}]\times {body\; weight}[g]}{{actual\; dose}[{Bq}]},$$

where the actual dose is corrected for radionuclide decay as a function of injected dose, uptake time, and radionuclide half-life,

2 $${actual\; dose}\left[{Bq}\right]={\left(\frac{{injected\; dose}\left[{Bq}\right]}{2}\right)}^{\frac{{uptake\; time}\left[s\right]}{{radionuclide\; half}-{life}\left[s\right]}}.$$

In addition, Python scripts for converting PET/CT data to NIfTI, mha, or hdf5 formats are also available from the FDG-PET/CT dataset preprocessing repository: https://github.com/lab-midas/TCIA_processing.

## Data Availability

This dataset is publicly available in DICOM format with a CC BY 4.0 license on The Cancer Imaging Archive (TCIA) under the collection name *PSMA-PET-CT-Lesions*^33^ (https://www.cancerimagingarchive.net/collection/PSMA-PET-CT-Lesions/). Further, it can be accessed in NIfTI format^34,35^. The full training dataset of the MICCAI autoPET III and IV Grand Challenges (containing Version 1 of the PSMA-PET/CT dataset) can also be downloaded in NIfTI format^42^.

## Code availability

We provide a repository for data preprocessing and conversion under https://github.com/ClinicalDataScience/tcia-psma-pet-ct-preprocessing. The custom dataset split file for training the baseline nnU-Net can be downloaded together with the NIfTI datasets^35,36^. Quantitative model evaluation was based on the metrics in https://github.com/lab-midas/autoPET/blob/master/val_script.py and https://github.com/lab-midas/autoPETV/blob/master/tutorials/metrics-tutorial.ipynb.
